# Evidence-based review of the management of early gastric cancer

**DOI:** 10.1093/gastro/got016

**Published:** 2013-05-06

**Authors:** Marissa Montgomery, Shinichi Fukuhara, Martin Karpeh, Steven Brower

**Affiliations:** Department of Surgery, Beth Israel Medical Center, New York NY, USA

**Keywords:** early gastric cancer, endoscopic mucosal resection, endoscopic submucosal dissection, gastrectomy

## Abstract

Although standard gastrectomy remains the most definitive locoregional treatment for early gastric cancer, it carries signiﬁcant perioperative morbidities. Surgical gastrectomy for resection of lymph nodes is not always required and endoscopic resection may be a treatment option for patients at negligible risk of lymph node metastasis. Furthermore, the criteria for endoscopic resection are expanding, along with the development of new technology, in both Eastern and western countries with high prevalence of early gastric cancer, where studies for endoscopic treatment modalities have been conducted. Within such a trend, however, it should be emphasized that early gastric cancer needs to be treated cautiously, especially in western countries, as several studies suggest that there may be differences in tumor biology and aggressiveness between Asian and non-Asian populations.

## INTRODUCTION

Early gastric cancer (EGC) is defined as invasive gastric adenocarcinoma that invades no more deeply than the submucosa—with or without lymph node metastasis irrespective of the tumor size [1]. The concept of EGC originated in Japan in 1962 [2]. The incidence of EGC varies depending on the population, although it has been increasing worldwide owing to advances in diagnostic techniques, resulting in an increased proportion of gastric cancers that are diagnosed at an early stage. In eastern Asia, up to one-half of resections for gastric adenocarcinoma represent EGC [3, 4]. In western countries, EGCs account for 15–21% of gastric adenocarcinomas [5–7]. This discrepancy has been postulated to be due to (i) nationwide screening programs instituted in eastern Asia [8], (ii) significantly higher incidence of gastric cancer in eastern Asia and (iii) differences in the interpretation of histology, compared with western countries [9]. Two major factors are associated with the prognosis of EGC: lymph node metastasis and depth of tumor invasion; however, only nodal involvement has been demonstrated to be an independent prognostic factor [10]. In EGC taken as a whole, nodal involvement is an infrequent event. Lymph node metastases are typically found in 6–15% of EGC, including 2–6% in mucosal cancer and 9–24% in submucosal cancer [10–12]. Advanced diagnostic and therapeutic endoscopic techniques are changing the paradigm of care in patients with EGC and literature out of eastern Asian countries has consistently reported promising results with endoscopic therapies for EGC. However, standard gastrectomy remains the most definitive locoregional treatment for EGC and endoscopic therapies are not recognized as first-line standard of care worldwide. By systematically reviewing the literature, this article focuses on recent developments and controversies relating to evidence-based treatment strategy in EGC.

## ENDOSCOPIC MANAGEMENT OF EGC

### Class I recommendations

–  In centers where endoscopic techniques are rarely used, gastrectomy remains the ‘gold standard’ for treatment of EGC. *Level of evidence: C*

### Class IIb recommendations

–  It is reasonable to treat early gastric cancers that meet the absolute indications established by the Japanese Gastric Cancer Association by utilizing endoscopic techniques at an experienced center. *Level of evidence: B*
–  Utilization of endoscopic submucosal dissection for early gastric cancers that meet the extended criteria for endoscopic management should be reserved for experienced endoscopists and for patients who agree to undergo long-term endoscopic surveillance. *Level of evidence: B*

With the excellent prognosis of EGC, which has a 5-year survival rate of greater than 85%, endoscopic therapies are becoming increasingly popular for treatment of EGC [5]. This is partially due to concern over subjecting patients to greater-than-necessary risk of morbidity from gastrectomy which, in some reports, is as high as 32% [12, 13].

A previous investigation, conducted in Italy, reported a series of 191 patients who underwent D2 resection for potentially curable gastric cancer [14]. In this series, overall morbidity and mortality rates were 20.9 and 3.1%, respectively; total gastrectomy had a higher mortality rate (7.46%) than distal gastrectomy (0.8%). Overall morbidity was shown to be similar to reports of non-radical gastrectomy or D1 procedures [15]. Several more recent studies have performed stage-matched comparisons of outcomes following both laparoscopic and open gastrectomies but are limited by small sample size [16–18]. A more recent retrospective cohort study, utilizing a large database, examined 9388 patients who underwent distal gastrectomy for early stage gastric cancer, either laparoscopic or open [19]. Similar mortality- and complication rates were observed in the laparoscopic and open distal gastrectomy groups; in-hospital mortality rates were 0.36 and 0.28%, respectively, and postoperative complications were 12.9 and 12.6%, respectively. Male gender, increased age and Charlson co-morbidity index were shown to be risk factors for postoperative morbidity [19].

Criteria for appropriate use of endoscopic therapy for the treatment of EGC have been outlined in the gastric cancer treatment guidelines published by the Japanese Gastric Cancer Association [20]. The indications are based on the principle that endoscopic therapy should be reserved for tumors having a size and morphology that are amenable for resection and that carry a very low probability of lymph node metastasis (LNM). These indications are categorized into two sets of indications: an absolute set of criteria for endoscopic therapy in its entirety (inclusive of endoscopic mucosal resection (EMR) and endoscopic submucosal dissection (ESD) and an expanded set of criteria for ESD as an investigational treatment. For the absolute indications, the tumor must meet all of the criteria listed in the guidelines, as shown in Table 1. The expanded criteria are a modified set, taking into account the improved resection capabilities of ESD, as compared with EMR, and are based more particularly on the principle of low likelihood of LNM. According to the Lauren classification, the histology type of gastric adenocarcinoma is classified as intestinal, diffuse, or mixed subtypes [21]. In general, intestinal gastric cancer is more likely to be sporadic than inherited and is related to environmental factors such as dietary habit and smoking, is typically located in the antrum and is associated with chronic inflammation and *helicobacter pylori* infection [22]. On the other hand, the diffuse type typically does not promote inflammation in the development of carcinogenesis. A molecular study for the diffuse type showed that CDH1 mutations and loss of expression of E-cadherin are seen in this type [23, 24].

**Table 1.** Criteria for endoscopic therapy (adapted from the Japanese Gastric Cancer Treatment Guidelines [20])

Absolute criteria (must fulfill all of the following)	Expanded criteria
Adenocarcinoma of differentiated histological type	cT1a plus one of the following:
No evidence of ulceration	Differentiated adenocarcinoma without evidence of ulceration ≤2 cm in diameter
Clinically assessed as T1a (cT1a) (confined to the mucosa)	Differentiated adenocarcinoma with evidence of ulceration but ≤3 cm in diameter
Diameter ≤2 cm	Undifferentiated type without evidence of ulceration and ≤2 cm in diameter.

The evidence for these recommendations is based on two Japanese studies investigating the LNM rates, each delineating specific subsets of EGC that carried negligible risk of lymph node metastases, as shown in Table 2. The first, conducted at the National Cancer Center Hospital and the Cancer Institute hospital in Japan, retrospectively reported a series of 5265 patients who had undergone gastrectomy with lymph node dissection for EGC [25]. They assessed nine clinico-pathological factors for predictability of LN involvement, including depth of invasion, histological type, size and presence of ulceration. They reported that, out of 1230 well- differentiated type lesions less than 3 cm in diameter—regardless of presence of ulceration— none were associated with metastases; none of the 929 lesions without ulceration were associated with metastases and none of the 145 differentiated carcinomas less than 3 cm in diameter, without lymphovascular invasion and with limited invasion into the submucosa, were associated with nodal metastases. For lesions greater than 3 cm, for both submucosal and intramucosal tumors, they demonstrated a correlation between size and lymphovascular involvement with nodal metastases.

**Table 2.** Descriptions of tumors found to have negligible risk of lymph node metastases

Gotoda *et al.* [25] (Total number of subjects: 5265)	Well-differentiated; <3 cm (0 of 1230)	Lesions without ulceration, regardless of other criteria (0 of 929)	Differentiated carcinomas <3 cm without lymphovascular invasion and limited invasion into submucosa (0 of 145)
Hirasawa *et al.* [26] (Total number of subjects: 3843)	Undifferentiated intramucosal lesions ≤2 cm, without lymphovascular invasions or ulcerative findings (0 of 310)		

The second study, conducted at the same institutions, focused specifically on prognostic factors for undifferentiated type lesions [26]. This series included 3843 patients with undifferentiated EGC, who had undergone gastrectomy with LN dissection. The overall incidence of LNM was 13.1%, with increased risk associated with invasion into the submucosa (23.8% with LNM in comparison with 4.9% of intramucosal lesions). Tumor size greater than 2.1 cm in diameter and lymphovascular invasion were also found to be independent predictors of LNM. Notably, in this study, none of the 310 undifferentiated intramucosal cancers ≤2 cm in diameter, without lymphovascular invasion or ulcerative findings, were demonstrated to have LNM.

While implementation of these criteria has become the standard of care in Japan and while there has been much analysis of the outcomes after EMR [12], there are few studies comparing long-term outcomes of EMR with gastrectomy, which is still the ‘gold standard’ for treatment of EGC in western countries. Probably the largest reported series was conducted in South Korea by Choi *et al.*, who performed a retrospective analysis of 551 patients who underwent either complete EMR or gastrectomy for EGC [27]. This study compared the outcomes of 172 patients who underwent EMR with 379 propensity-matched patients who underwent gastrectomy. The EMR technique used in this study consisted of diluted epinephrine submucosal injection and circumferential pre-cutting, followed by snare resection. Resection was considered complete when the tumor could be removed either *en bloc* or piecemeal with post-removal reconstruction, with tumor-free lateral and vertical margins and with no evidence of lymphovascular invasion. Routine follow-up for EMR consisted of endoscopy at 6 months, 1 year and annually thereafter. The primary endpoints observed were death and tumor recurrence. The median follow-up period was 81 and 88 months for EMR and gastrectomy, respectively. Risk of death was not found to be significantly different between the two groups and there was no significant difference in the risk of recurrence during the follow-up period. Only two patients (1.2%) in the EMR group had recurrences; one had a local recurrence, diagnosed pathologically at the resection margin, and the other had a regional recurrence in the regional gastric lymph nodes. The local recurrence was treated successfully with repeat EMR and the regional recurrence was treated by surgery. There was no significant difference in overall survival between recurrences in the EMR group versus the gastrectomy group. EMR was also found to be associated with a higher risk of metachronous gastric cancer (5.8 vs 1.1% in the surgery group) which the authors attributed to the residual gastric mucosa probably containing high-risk areas, such as mucosa with atrophic gastritis and intestinal metaplasia. All metachronous EGCs in this study were treated successfully with either EMR or surgery, resulting in no difference in overall survival. Complication rates were found to be similar in both the EMR and surgery groups.

There is also fairly limited evidence concerning ESD versus gastrectomy, although this is not unexpected, given the recent development of this technique and resultant shortage of data on long-term outcomes. A recent retrospective study conducted in Hong Kong provides the most recent evidence for comparison of ESD against gastrectomy, with analysis of 114 patients with either severe dysplasia or EGC [13]. All patients underwent endoscopic ultrasound and image-enhanced endoscopy prior to either procedure. ESD was performed by one of three endoscopists utilizing submucosal injection of saline/epinephrine/carmine, followed by dissection between the mucosa and *muscularis propria* utilizing the insulated tip knife, hook knife or triangular tip knife. Gastrectomy consisted of a D1 + B resection, either open or laparoscopic. Follow-up for ESD consisted of routine endoscopy at 3-month intervals for 2 years, then 6-month intervals until 5 years after the procedure. A total of 40 patients underwent radical gastrectomy, with 74 patients receiving treatment with ESD. ESD was successful (obtaining *en bloc* resection) in 68 of 74 cases. The overall complication rate was higher in the gastrectomy group, although the study found no significant difference between the groups in the 3-year survival rate.

There is also limited data comparing ESD with EMR. To date, there is no randomized, controlled trial comparing these endoscopic techniques. A meta-analysis conducted recently found only 12 studies appropriate for comparison of ESD with EMR and all were either non-concurrent cohort studies or retrospective cohort studies [28]. This meta-analysis found an increase in efficacy in the pooled data, with significantly higher complete resection rates and lower recurrence rates than found in the EMR group. The mortality risk between ESD and EMR was not significantly different, although they discovered a significantly higher perforation rate with ESD.

In cases of recurrence or incomplete resection, either repeat endoscopic resection (in cases of local recurrence) or gastrectomy is indicated [12, 25, 29, 30]. Close follow-up with endoscopic surveillance and computed tomographic (CT) scanning is universally required for all patients, including those undergoing complete resection. There is some concern regarding a delay in diagnosis as a result of performing endoscopic therapy as a primary treatment, as this necessarily delays the time to gastrectomy in patients who have incomplete resection and delays diagnosis for those who may develop lymph node metastasis. One study by Lee *et al.* retrospectively studied 13 patients who required gastrectomy following an incomplete endoscopic resection by either EMR or ESD [29]. They discovered three out of the 13 cases to have positive lymph node metastasis; two of the cases had histological lymphatic invasion and all three had submucosal invasion. The authors concluded that all patients with incomplete resection should undergo gastrectomy—and not a repeat endoscopic procedure—due to the high risk of lymph node metastasis [29]. Another study by Goto *et al.* described 31 subjects who underwent gastrectomy following ESD; indications in this case were specific histopathological criteria in the endoscopic specimen, which resulted in exclusion from the expanded criteria for ESD [30]. This study aimed to assess whether there was any negative effect on patient outcome through performing ESD prior to gastrectomy, since this would constitute a delay in curative treatment for these patients. Despite finding that four of the 31 patients had lymph node metastases, all patients requiring additional gastrectomy had recurrence-free survival with a median follow-up of 3.4 years. These studies are limited by small numbers and lack of patient diversity (both studies were conducted in eastern Asia). In fact, there is a paucity of published material concerning the possible effect of delayed diagnosis of lymph node metastases, possibly due to the fact that, if the absolute guidelines for utilization of endoscopic treatment for EGC are followed, the risk of lymph node metastases utilizing the absolute criteria is essentially zero in certain Asian populations [25, 26].

These studies suggest that endoscopic therapy may be appropriate for a select cohort of patients who meet standardized criteria, based on a low likelihood of nodal invasion. However, it should be emphasized that most data concerning these treatments originates from a few institutions in countries where gastric cancer carries a significantly higher incidence than may be seen worldwide and the evidence may be limited, due to prognostic factors such as race and institution experience. In fact, there have been several reports suggesting significant ethnic and racial differences in incidence and survival of gastric cancer. According to these studies, Asian patients consistently enjoy increased survival rates, compared with their western counterparts, even in studies conducted in the United States, where same-treatment strategies were provided in each group [31]. This survival rate advantage may reflect less aggressive tumor biology in an Asian population, although the prognostic significance of ethnicity specifically for EGC is as yet unclear. If the biologies of gastric cancer in patients of Asian and non-Asian populations are truly different, clinical data utilizing a study population exclusively comprising Asian patients, obtained in eastern Asian countries, may simply not be applicable to patients in western countries. Additionally, *en bloc* resection utilizing either ESD or EMR is a technically difficult task and requires extensive experience specific to these techniques, and may not be feasible in countries which have a relatively low incidence of gastric cancer. We believe that more extensive investigation of these discrepancies between Asian and non-Asian groups is crucial for the application of an appropriate of treatment strategy in each group.

## SURGICAL MANAGEMENT OF EGC

### Class I recommendations

–  Sub-total gastrectomy—as compared with total gastrectomy—is sufficient for curative resection of distal early gastric cancers. *Level of evidence: A*

### Class IIb recommendations

–  Laparoscopy-assisted distal gastrectomy should be reserved for patients whose clinical staging carries a low likelihood of lymph node metastases. *Level of evidence: B*
–  D2 resections should be used sparingly in early gastric cancers, which carry a low likelihood of nodal metastasis. *Level of evidence: C*

Surgical resection remains the ‘gold standard’ for treatment of all potentially curable gastric cancers. Adequate resection of both gross and microscopic disease, as well as resection of any lymphatic or vascular invasion, is necessary for resection with curative intent. However, there is currently no consensus as to stage-directed surgical management specifically for EGC, and controversy remains concerning the appropriate extent of gastric resection (either distal or total), the extent of lymph node dissection, the surgical modality (laparoscopically assisted versus open) and the role of adjacent organ resection. These areas of controversy may not apply to a certain subset of EGCs that have been shown to carry negligible risk of nodal metastasis or extensive nodal invasion [25, 26], however evidence of this negligible risk remains applicable only to the eastern Asian countries where these studies were conducted and, in western countries, the data concerning likelihood of nodal metastasis is lacking.

The extent of gastric resection, lymph node dissection and the matter of surgical modality are controversial, owing to the significant impact of an R0 resection on gastric cancer prognosis. An early, prospective, multicenter observation trial was conducted in Germany in 1986 and followed a total of 1654 patients with gastric cancer, who underwent either D1 or D2 gastric resections with curative intent [32]. The 10-year survival was calculated to be 36.1% for those patients who had undergone an R0 resection versus 26.3% across the entire study population. This particular study included patients with EGC; however, they were only a portion of the total patient population and patients were not stratified according to pre-operative clinical staging and the extent of nodal dissection only displayed an effect on pathological Stage II tumors. To date there has not been a study conducted outside of eastern Asia, to delineate the effect of R0 resection, in comparison to R1 resection, specifically for early gastric cancers.

Optimal extent of gastric resection, either distal or total gastrectomy, has been analysed in three prospective clinical trials. Gouzi *et al.* conducted a multicenter, prospective, randomized trial in France in the early 1980’s, examining the 5-year survival rates in a series of 169 patients with resectable antral carcinoma [33]. A total of 76 patients were randomized to the ‘total gastrectomy’ group and 93 in the ‘sub-total gastrectomy’ group; approximately half of each group (53 and 55%, respectively) had well-differentiated histology and 42% of each group exhibited superficial tumors without serosal invasion. There was no difference in five-year survival between the sub-total gastrectomy- and total gastrectomy groups, although serosal extension and lymph node involvement were both shown to have a significant negative effect on survival. While the concept of EGC was not defined during this study, early-stage tumors were present in a large portion of the patient population. An additional prospective, randomized clinical trial was conducted to examine the comparative efficacy of an R1 sub-total gastrectomy versus R3 total gastrectomy with splenectomy and distal pancreatectomy [34]. A total of 19 patients with early gastric cancer were included in the study, with 10 patients undergoing an R1 resection and nine undergoing an R3 resection. Three of the 19 patients with T1 disease were also found to have nodal metastases. Overall analysis, inclusive of all stages, resulted in an increase in survival resulting from an R1 sub-total gastrectomy, with a median survival of 1511 days, versus 922 days for the R3 resection. Although subset analysis was not conducted for the early gastric cancer group, the improved survival seemed to be related to the morbidity of the R3 resection, which would be consistent regardless of the stage of the tumor. The third multicenter, randomized, controlled trial was conducted in Italy and evaluated a series of 618 patients who underwent either sub-total or total gastrectomy [35]. This study, which also included early gastric cancers, concluded that the five-year survival was equivalent and recommended that the preferred extent of resection should be sub-total gastrectomy, due to the improved quality of life and decreased morbidity when compared with a total gastrectomy. Overall, these studies suggest that sub-total gastrectomy is sufficient for curative resection of gastric cancer inclusive of early gastric cancer.

Laparoscopy-assisted distal gastrectomy (LADG) was first introduced by Kitano *et al.* in 1994 and has since been the topic of much controversy [34]. The most comprehensive review of the evidence comparing LADG against open distal gastrectomy—particularly for early gastric cancer—has recently been published by Zeng *et al.* [37]. They identified a total of 22 studies for inclusion, five of which were randomized clinical trials, for a total of 3411 participants, with 1596 who underwent LADG and 1815 who underwent open distal gastrectomy (ODG). The extent of lymph node dissection was variable, with either D1 or D2 resections being performed during both LADG and ODG procedures. They determined that the mean number of lymph nodes retrieved in LADG was similar to those obtained in ODG. The conclusion of this study was that the currently available evidence was insufficient to discount a survival benefit from ODG and the recommendation was made that ODG should be considered in patients who have a high likelihood of lymph node metastasis. Notably, all of the studies included in this meta-analysis originated from eastern Asian countries—specifically Japan, Korea and China—where gastrectomy is typically reserved for early gastric cancers that do not meet criteria for endoscopic therapy. Thus, the available evidence is skewed by the fact that early gastric cancers that have lower likelihood of nodal metastases are not included, due to an alternate method of treatment—namely EMR—which may not be a first-line standard of care worldwide.

The most controversial aspect of the surgical management of EGC is perhaps the determination of adequate lymph node dissection, a factor that plays a crucial role in both of the previous areas of controversy: extent of surgical resection and efficacy of LADG. Extent of lymph node dissection has been categorized by the Japanese Gastric Cancer Association as shown in Table 3, with definitions of D1 and D2 dissections varying according to which type of gastrectomy is performed. Indications for extent of dissection from the same guidelines have also been listed. EGC may qualify for either D1, D1+ or D2 lymph node dissection. While these guidelines are based on extensive evidence from Japanese studies, western studies have not been able to provide sufficient evidence for their worldwide application. A retrospective review conducted utilizing data from the American College of Surgeons analysed the outcomes of 3804 patients who underwent curative resection for gastric cancer with either D1 or D2 lymph node dissection [38]. D2 lymph node dissection did not significantly improve survival, resulting in a 5-year survival rate of 26.3% in comparison to the 30% associated with a D1 resection. However, this study did not report subgroup analysis specific to early gastric cancer. Two prospective, randomized trials were conducted in western Europe to further investigate the efficacy of extended lymph node dissection: the Dutch Gastric Cancer Group trial and the Medical Research Council trial in the UK [39, 40]. Neither trial demonstrated a survival advantage in D2 resection. In both trials, the subjects were randomly assigned to either a D1 or a D2 gastrectomy, irrespective of clinical stage. Subgroup and multivariate analyses were performed in both studies to isolate the influence of individual staging characteristics: however, since the Japanese guidelines for treatment are based on strict clinical staging criteria, multivariate analysis may be insufficient to evaluate treatment recommendations, which are based on probabilities of metastases assigned to very specific clinical stages.

**Table 3.** Lymph node dissection (adapted from the Japanese Gastric Cancer Treatment Guidelines [20])

	Definition for total gastrectomy	Definition for distal gastrectomy	Indication
D1	Resection of lymph node levels 1–7	Resection of lymph node levels 1, 3, 4sb, 4d, 5, 6 and 7	T1a tumors that do not meet criteria for endoscopic therapy and for cT1bN0 differentiated type lesions ≤1.5 cm
D1+	Resection of lymph node levels 1–7, 8a and 9	Resection of lymph node levels 1, 3, 4sb, 4d, 5, 6, 7, 8a and 9	cT1N0 tumors other than above
D2	Resection of lymph node levels 1–7, 8a, 9, 11p and 12a	Resection of lymph node levels 1, 3, 4sb, 4d, 5, 6, 7, 8a, 9, 11p and 12a	Potentially curable T2–T4 tumors; cT1N+ tumors

## SUMMARY

Development of evidenced-based treatment strategies for early gastric cancer continues to be a challenge. The plethora of data out of eastern Asia, a result of the recognition of the high incidence of gastric cancer and endoscopic—as well as surgical—experience in these countries, has allowed for the formation of well-established guidelines, such as the Japanese Gastric Cancer Treatment Guidelines. The evolution of these disease-specific guidelines is certainly the model for further early gastric cancer research, beginning with studies examining the likelihood of lymph node metastases based on clinical diagnosis of early gastric cancer, utilizing this evidence to support less-invasive treatments for certain subsets of qualifying tumors and, finally, randomized trials to compare proposed treatments with standards of care. In comparison, the prevalence of gastric cancer in western countries is significantly lower and shows some evidence of significantly different tumor biology, making it more difficult to power a quality, randomized study or to provide a large patient database to mirror the Japanese experience of predicting lymph node metastases based solely on clinical staging. For this reason, caution should be taken when applying this evidence outside eastern Asia and the less-invasive modalities (EMR/ESD) should not be considered as standard treatments until there is evidence that EGC outside east Asia carries a similarly negligible risk of nodal metastases.

**Conflict of interest:** none declared.

## APPENDIX 1

- Level of evidence A: recommendation based on evidence from multiple randomized trials or meta-analyses
- Level of evidence B: recommendation based on evidence from a single randomized trial or non-randomized studies
- Level of evidence C: recommendation based on expert opinion, case studies or standards of care
- Class I: conditions for which there is evidence and/or general agreement that a given procedure or treatment is useful and effective
- Class II: conditions for which there is conflicting evidence and/or a divergence of opinion about the usefulness/ efficacy of a procedure or treatment
- Class IIa: weight of evidence/opinion is in favor of usefulness/efficacy
- Class IIb: usefulness/efficacy is less well established by evidence/opinion
- Class III: conditions for which there is evidence and/or general agreement that the procedure/treatment is not useful/effective and in some cases may be harmful
